# Toxoplasmosis in the outer retina

**DOI:** 10.22336/rjo.2024.37

**Published:** 2024

**Authors:** Ajla Pidro Miokovic, Mirko Ratkovic, Aida Pidro Gadzo

**Affiliations:** * Polyclinic Vukas, Zagreb, Croatia; ** Department of Ophthalmology at NU Sjukvarden, Uddevalla Sjukhus, Sweden; *** Ophthalmology Department, “Prim. Dr. Abdulah Nakaš” General Hospital, Sarajevo, Bosnia and Herzegovina

**Keywords:** uveitis, ocular inflammation, toxoplasmosis, optical coherence tomography

## Abstract

**Objective:** To present a case of ocular toxoplasmosis.

**Materials and methods:** A sixteen-year-old female patient presented to our clinic with complaints regarding decreased vision in her right eye (BCVA 0.5), starting five days before the exam. Her anamnestic data revealed a previous history of ocular toxoplasmosis in her left eye. OCT scans of the inner retina identified a huge cystic space, located posterior to the inner line, off the outer plexiform layer, with a small amount of hyperreflective foci. Other features of OCT included membranous-like structures on inner borders and elongation and splitting of the inner segment/outer segment junction. In later stages, beginning signs of retinitis and scaring could be observed.

**Results:** The patient was treated with sulfamethoxazole/trimethoprim and prednisolone. After two weeks, total regression occurred and visual acuity and OCT remained stable for 6 months (BCVA 1.0).

**Discussion:** Ocular toxoplasmosis can cause significant vision loss due to retinitis and scarring. Following treatment with sulfamethoxazole/trimethoprim and prednisolone, the patient’s condition improved significantly and her visual acuity remained stable.

**Conclusion:** On clinical examination and using OCT, rare morphological cystoid spaces (CS) can be identified as huge outer retina cysts (HORC), which are pathognomonic for posterior uveitis.

**Abbreviations:** HORC = huge outer retinal cyst, OCT = optical coherence tomography, BCVA = best corrected visual acuity, CS = cyst space, OPL = outer plexiform layer, HRF = hyper reflective foci, RPE = retinal pigment epithelium, IS = inner segment, OS = outer segment, ERM = epiretinal membrane, PORT = punctate outer retinal toxoplasmosis, ELM = external limiting membrane

## Introduction

Ocular toxoplasmosis, the most common cause of infectious posterior uveitis [**1**], typically presents as whitish-gray retinitis and vitritis above the lesion [**2**,**3**]. Cystoid changes are rare and severe complications [**4**,**5**], occurring at the level of cystoid macular edema 7.5%, cystoid degeneration 15%, and a huge cyst located in the outer retina (HORC) 2.5% [**6**]. The technology advances enable layer-by-layer evaluation, including a good visualization of all microstructures in the neurosensory retina [**7**].

This paper reported right-eye ocular toxoplasmosis and its unusual presentation as a large cyst located in the outer retina, and a follow-up of cystoid changes seen on macular OCT.

## Case report

A 16-year-old female patient, previously diagnosed with left-eye ocular toxoplasmosis, presented with a complaint of decreased vision in the right eye for 5 days. The best corrected visual acuity (BCVA) of the right eye was 0.5 on presentation, compared to previous reports, where it was 1.0. The BCVA of the left eye was 0.05 and corresponded to her anterior reports. Other findings were unremarkable on both eyes. Fundus examination revealed a macular scar with no activity signs on the left eye. The patient presented multifocal gray-white lesions on the right eye, located at the deep retina and retinal pigment epithelium, a small pigmented macular lesion consistent with small scaring, and mild vitritis.

**Fig. 1:**
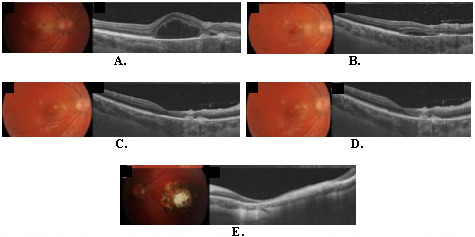
Fundus photograph and OCT scans. Right eye: at presentation (**A**); at one week (**B**); at two weeks (**C**); at 6 months (**D**); Left eye: at presentation and through follow-up (**E**)

OCT scans of the inner retina identified a huge cystic space (CS), with adjacent smaller CS. The term used to identify this kind of cystic change was the huge outer retina cyst (HORC), located posterior to the inner line of the outer plexiform layer (OPL). The highest point of the cyst was 475 microns. A small amount of hyper-reflective foci (HRF) was observed on the presentation day inside the HORC. A membranous-like structure was identified on the borders of the cysts and laid on the retinal pigment epithelium (RPE). The elongation and splitting of the inner segment/outer segment junction (IS/OS) and its disruption and outer segment (OS) irregularity were observed and described. An area of active retinitis was observed in the nasal portion from HORC. Beginning signs of scaring on the OCT were identified in the nasal portion, underneath retinitis: RPE was irregularly bunched, with visualization of underlying Bruch’s membrane. All stages are visible in **Fig. 1 A-E**.

The diagnosis of ocular toxoplasmosis was clinically made and the patient was treated with sulfamethoxazole/trimethoprim 800/160 mg (Bactrim DS) twice a day for four weeks. In addition, Prednisolone was given in a tapering dose regimen of 20 mg → 10 mg → 5 mg → 2.5 mg (7 days tapering). The Sabin and Feldman reactions were done 7 days after the therapy administration; IgG-AT measured 3.49, and IgM-AK was negative. On follow-up examination, 7 days after the beginning of BCVA therapy, the right eye was 0.75, and, two weeks after, it was 1.0, and remained stable for 6 months after. Differential diagnosis also included Vogt-Koyanagi-Harada Disease and Acute Posterior Multifocal Placoid Pigment Epitheliopathy [**8**].

Signs of vitritis were higher on the second visit, but after 4 weeks it was also resolved. On the final visit, normal fovea architecture was observed, with somewhat thinning and hypo-reflectivity of the IS/OS junction, consistent with good visual acuity. A small amount of membranous hyperreflective material above RPE was present, losing reflectivity with time.

The external limiting membrane could be identified on any of the OCT scans around HORC but was identified in areas outside of HORC, and again in the whole retina after the resolution or retinitis, even where HORC existed. HORC was much smaller after 7 days of treatment and resolved after 2 weeks.

At that point, a mound of deep hyper-reflective material within the zone of RPE atrophy was present in the region of previously active retinitis. A bulb-shaped structure was described with the RPE disruption and Bruch’s membrane. In the inner part of the retina, atrophy persisted with a hyper-reflective membrane (secondary ERM), with thickened and detached posterior hyaloid, making this the final step in the acute phase of ocular toxoplasmosis.

## Discussion

In this case, ocular toxoplasmosis presented as a multifocal necrotizing chorioretinitis. The main goal of the treatment was to stop parasite multiplication and reduce the damage due to necrosis of chorioretinal tissue [**9**,**10**]. Our patient had sight-threatening retinitis so the treatment included antibiotics and oral corticosteroids. The therapy showed quick response with both visual and anatomical improvement. Therapy showed quick response with both visual and anatomical improvement. The reason for low IgM levels was that testing was done in a later phase, 7 days into treatment.

Atypical OT presentations can raise a challenge in the diagnostics and delay treatment, resulting in poor vision recovery [**11**]. The group of immunocompromised and elderly patients can present much more severe atypical OT presentations with multiple lesions.

In this case, we observed a very unusual presentation, the Punctate Outer Retinal Toxoplasmosis (PORT), with a specific huge cystic space in the outer retina (HORC). HORC can be identified in only 2,5% of ocular toxoplasmosis patients, making it the rarest cystic presentation in this chorioretinal disease [**6**]. We observed HORC in the acute phase, and its regression after the therapy was administered.

The cyst’s location was unclear, but our findings supported the hypothesis that it was in the retina and not underneath it or RPE. More precisely, it was between the ELM and the inner boundary of the RPE [**6**]. A membranous hyperreflective material inside of HORC is considered an inflammatory response and somewhat causes the thickening of the PR layer [**12**]. The residual of this structure after HORC, in the form of a slight hyper-reflective line on the RPE, which diminished with time and remission of inflammation, was additional proof of this theory.

To our knowledge, the only clinical report of HORC presence in the photoreceptor layer (OS) in ocular toxoplasmosis was by Ouyang et al. [**6**]. These findings of ocular toxoplasmosis confirm similar studies on animal models, describing the enlargement of the interstitial space in PR and cystoid changes in the outer plexiform layer [**13**].

Lujan argued that HORC is not a cyst in the outer retina, but rather subretinal fluid [**14**]. Arguments are found in the anatomical lack of space and visualization of ELM in cases where some regression in HORC was observed already [**14**]. Few studies evaluated cystic spaces in toxoplasmosis; but, if we expand our interest to other forms of posterior uveitis, we can find many papers on edema in the PR layer in Vogt-Koyanagi-Harada [**15**]. Lee et al. consider acute inflammation the cause of swelling of the photoreceptors. They argue that the disc of cones can be unfolded without rupturing the membrane since they are made of it. Afterward, the edema can progress to the junction of the inner and outer retina to the distal portions, leaving the ELM invisible in acute phases. The changes in photoreceptors can be reversible and irreversible. After therapy, inflammation is reduced and the reversible portion folded back into place, but, if the photoreceptors are damaged and left behind in the RPE phagocytosis, they can continue after the inflammation is controlled, thus, those segments will be removed [**15**]. This mechanism can explain residual tissue on top of RPE, with a similar thickness to the outer segment layer of the normal retina. This process can be observed while regaining normal layering of the retina and in remission. Only subretinal fluid can be observed, with ELM again visible above it. All can be applied in the case of HORC in toxoplasmosis, as presented in our case report.

Also, the damage from edema after normal architecture restoration can be observed in the changes in PR density. The mean cone densities in areas with a previous presence of cystoid spaces were significantly lower than those without cystoid spaces at the baseline [**16**]. In our case, it was confirmed by less hyperreflectivity of the outer retina structures on scans, during the follow-up period. More research has to be done to identify the time at which photoreceptors can maintain full function even elongated and if the cyst’s volume can be a factor that influences it.

## Conclusion

With the appropriate use of therapy, the visual prognosis for patients with atypical forms of ocular toxoplasmosis may be good, as shown in this case. The presence of cystoid spaces might be a sign of possible damage to cone photoreceptors if not treated on time, and prompt treatment is needed in this atypical form.

**Conflicts of Interest Statement**

The authors declare no conflict of interest.

**Informed Consent and Human and Animal Rights Statement**

Informed consent has been obtained from the patient included in this study.

**Authorization for the use of human subjects**

Ethical approval: The research related to human use complies with all the relevant national regulations, and institutional policies, as per the tenets of the Helsinki Declaration, and has been approved by the review board of “Polyclinic Vukas, Zagreb, Croatia (0207-805, 20.12.2023.

**Acknowledgments**

None.

**Sources of Funding**

None.

**Disclosures**

None.
